# Overlap syndrome of autoimmune hepatitis and primary biliary cholangitis complicated with atypical hepatocellular carcinoma: a case report

**DOI:** 10.1186/s13256-023-03932-y

**Published:** 2023-07-25

**Authors:** Teodora Surdea-Blaga, Roxana L. Cărăguț, Cosmin Caraiani, Zeno Spârchez, Nadim al Hajjar, Dan L. Dumitrașcu

**Affiliations:** 1 “Iuliu Hațieganu” University of Medicine and Pharmacy, Cluj-Napoca, Romania; 2 2nd Department of Internal Medicine, County Emergency Hospital, Cluj-Napoca, Romania; 3 Regional Institute of Gastroenterology and Hepatology, No. 19-21 Croitorilor Street, 400162 Cluj-Napoca, Cluj Romania

**Keywords:** Hepatocellular carcinoma, Primary biliary cholangitis, Autoimmune hepatitis, Liver cirrhosis, Atypical liver resection, Case report

## Abstract

**Background:**

Hepatocellular carcinoma (HCC) is a primary tumor of the liver. The majority of HCCs are associated most frequently with chronic B or C viral hepatitis, alcohol intake or aflatoxin exposure. Cirrhosis is a strong risk factor associated with HCC. The causes of liver cirrhosis are chronic viral hepatitis, alcohol intake, metabolic diseases (NAFLD), hemocromathosis, alfa 1 antitrypsisn deficiency. All aetiologic forms of cirrhosis are at risk to be complicated by HCC development, but the risk is higher for patients diagnosed with chronic viral hepatitis. Comparing to the above-mentioned causes, PBC and AIH are less associated with the risk of HCC development.

**Case summary:**

A 71-year old Caucasian female previously diagnosed with overlap syndrome (AIH type 1 and PBC—ANA, SMA and AMA antibodies positive), liver cirrhosis, a nodule in the VI/VIIth hepatic segment, systemic sclerosis sine scleroderma, Hashimoto's thyroiditis, antiphospholipid syndrome, gastric antral vascular ectasia (GAVE) (with 2 previous sessions of argon plasma coagulation), cholecystectomy, arterial hypertension and nephro-angiosclerosis presented to the 2nd Department of Internal Medicine in Cluj-Napoca for a follow-up. The patient was following treatment with UDCA (Ursodeoxycholic acid), azathioprine, Plaquenil, calcium channel blockers, angiotensin-converting-enzyme inhibitor, calcium and vitamin D supplementation. The abdominal ultrasound showed a subcapsular hypoechoic nodule with a diameter of 29 mm (at the moment of the diagnosis the diameter was 9/10 mm) in the VI/VIIth hepatic segment. The contrast-enhanced ultrasound (CEUS) characterised the nodule as specific for hepatocellular carcinoma (LI-RADS 5). On MRI with gadoxetate disodium the nodule was hypovascular, non-specific, being classified as LI-RADS 3. An atypical resection of the VIIth hepatic segment was performed and the histohistological examination and imunohistochemistry (Hep Par-a positive, Glypican3 positive, CD34 positive) revealed a moderately differentiated hepatocellular carcinoma (G2), pT2 N0 M0 L0 V1 R0.

**Conclusion:**

Autoimmune hepatitis, PBC and the overlap syndrome are less associated with the development of liver cirrhosis and HCC than other chronic liver diseases, especially if other risk factors are not associated. This case highlights the importance of a proper surveillance of cirrhotic patients every 6 months including abdominal ultrasound and AFP levels is crucial for an early diagnosis of a HCC.

## Introduction

Primary Biliary Cholangitis (PBC) is an uncommon cholestatic, chronic, autoimmune disease. The global incidence rate is between 0.33 and 0.58 per 100,000 inhabitants/year and the prevalence varies from 1.91 to 40.2 per 100,000 individuals [1]. In Europe, the estimated incidence is 1–2 per 100,000 inhabitants/year [2]. Women are more affected by PBC, with a female: male ratio of 10:1 [3]. PBC is characterized by the presence of antimitochondrial antibodies (AMA) in 90–95% of the cases or antinuclear antibodies (ANA) in 50% of the cases. The histological characteristic is a chronic, non-suppurative, lymphocytic and granulomatous cholangitis, affecting mainly the small bile ducts [2]. The most frequent symptoms are cholestatic pruritus, fatigue, abdominal discomfort, sicca complex, sleeplessness, cognitive dysfunction, and depression. These symptoms affect the patient’s quality of life [2, 4]. A main aspect of PBC is the presence of cholestasis. Cholestasis can manifest with pruritus, fatigue, jaundice, and abdominal discomfort in the right upper quadrant. Elevated serum levels of gamma-glutamyltranspeptidase (GGT), alkaline phosphatase (ALP) and direct bilirubin (advanced stages) are characteristics of cholestasis [5].

PBC-Autoimmune hepatitis (AIH) overlap syndrome’ is diagnosed in about 8 to 10% of the patients initially diagnosed with PBC [6].

Paris diagnostic criteria for PBC-AIH overlap syndrome are summarized in Table 1.

**Table 1:** Paris diagnostic criteria for PBC-AIH overlap syndrome. Adapted to European Association for the Study of the Liver. EASL Clinical Practice Guidelines: management of cholestatic liver diseases. J Hepatol. 2009 Aug;51(2):237–67. https://doi.org/10.1016/j.jhep.2009.04.009

At least 2 of the following:	And at least 2 of the following:
ALP > 2 × ULN (upper limit of normal) or GGT > 5 × ULN	ALT (alanine aminotransferase) > 5 × ULN
AMA > 1:40	IgG (Immunoglobulin G) serum levels > 2 × ULN or smooth muscle autoantibody (SMA) +
Histology: florid bile duct lesion	Histology: moderate or severe interface hepatitis

*ALP* alkaline phosphatase, *AMA* anti-mitochondrial antibodies, *ALT* alanine aminotransferase, *IgG* Immunoglobulin G, *SMA* smooth muscle autoantibody, *ULN* upper limit normal

Liver biopsy is considered essential in clinical practice to confirm the presence of AIH and can be considered in patients diagnosed with PBC who have disproportionate elevations of IgG and/or ALT [2, 5].

Double stranded DNA autoantibodies, antibodies against soluble liver antigen (SLA) or liver pancreas (LP) have also been associated with AIH in patients initially diagnosed with PBC.

Patients with PBC and characteristics of AIH can benefit from immunosuppressive treatment along with ursodeoxycholic acid (UDCA). Immunosuppressive treatment is recommended in patients with severe interface hepatitis, and it is taken into consideration in patients with moderate interface hepatitis [2].

Hepatocellular carcinoma (HCC) is a known consequence of AIH related cirrhosis but its occurrence is considerably less frequent compared to most other causes of liver cirrhosis, even PBC. HCC development in PBC is lower compared to other causes of liver cirrhosis (chronic viral hepatitis, alcohol intake, metabolic diseases (NAFLD), hemocromathosis, and so on) but the risk of occurrence is increased by the presence of advanced disease in patients who are UDCA non-responders and in men rather than women [6, 7].

## Case report

### Chief complaints

In June 2021 a 71-year Caucasian old female presented in the 2nd Department of Internal Medicine Cluj-Napoca without any complaints, in order to reevaluate a subcapsular hypoechoic nodule located in the VI/VIIth hepatic segment detected during an abdominal ultrasound in October 2020. The patient didn’t have any symptoms.

### History of present illness

In October 2020 during an abdominal ultrasound in the VI/VIIth hepatic segment it was detected a subcapsular hypoechoic nodule with a diameter of 9/10 mm. Given the size of the nodule at the time, a follow-up examination was recommended after 3 months, but due to the COVID-19 pandemic the patient did not follow this recommendation.

### History of past illness

The disease was diagnosed in 2014 when the patient was admitted for high values of arterial blood pressure, severe fatigue and pallor was found at the physical exam. Laboratory findings showed severe microcytic anemia (hemoglobin of 7 g/dl), increased transaminases (AST = 122U/L, ALT = 74 U/L), and increased GGT (126 U/L). Abdominal ultrasound revealed enlarged and slightly inhomogeneous liver and enlarged spleen. Upper gastrointestinal (GI) endoscopy revealed some antral vascular ectasia, while lower GI endoscopy was normal. During the diagnostic workup for chronic liver diseases, HBs antigen and HCV antibodies were negative, ferritin was low, ANA, SMA and AMA antibodies were positive with titers of 1/640, 1/320 and 1/320 respectively. The diagnosis of overlap syndrome (AIH type 1 and PBC) was established. To confirm the diagnosis, a liver biopsy was performed at the time. The result of the histological exam, which included 11 portal spaces, confirmed the overlap syndrome, showed severe interface hepatitis, proliferation of the bile ducts, fibrotic changes with rare regenerative nodules, suggestive for cirrhosis. Given the severe interface hepatitis, increased transaminases and increased values for IgG (35.8 g/L, 2.2 × ULN), and IgM (4.62 g/L, 2 × ULN), the induction of remission with prednisone (40 mg/day) was started. Azathioprine (1.5 mg/kg/day) and UDCA (15 mg/kg/day) were also initiated. The prednisone was tapered in 3 months to 15 mg/day. There were several attempts to reduce further the dose of Prednisone, but transaminases had increased every time. In 2017, while transaminases were within normal range, the corticosteroid was stopped, and the patient continued only on UDCA and azathioprine. The patient had regular follow up visits in our department, every 6 months, laboratory tests, including alpha fetoprotein and abdominal ultrasound were performed on every visit. The Fibroscan performed in 2019 showed a hepatic stiffness of 5.2 kPa, corresponding to F1 fibrosis.

### Personal and family history

The patient was also diagnosed in the past with systemic sclerosis sine scleroderma, Hashimoto's thyroiditis, antiphospholipid syndrome, gastric antral vascular ectasia (GAVE) (with 2 previous sessions of argon plasma coagulation), arterial hypertension and nephro-angiosclerosis.

Surgical history: cholecystectomy (1998).

The patient’s family history was unremarkable.

### Physical examination

The patient’s blood pressure was 125/80 mmHg at admission, a heart rate of 82 bpm, the temperature was normal (36,4 °C) and a SpO2 of 97%. The physical examination was normal, without any pathological element.

### Laboratory examinations

Blood analysis revealed a increased value of total bilirubin 2.05 mg/dL, direct bilirubin of 0.40 mg/dL, creatinine 1.01 mg/dL and urea 51.43 mg/dL. The AFP level was slightly increased 10.8 ng/ml (ULN = 9 ng/ml).

### Imaging examinations

**Fig. 1:**
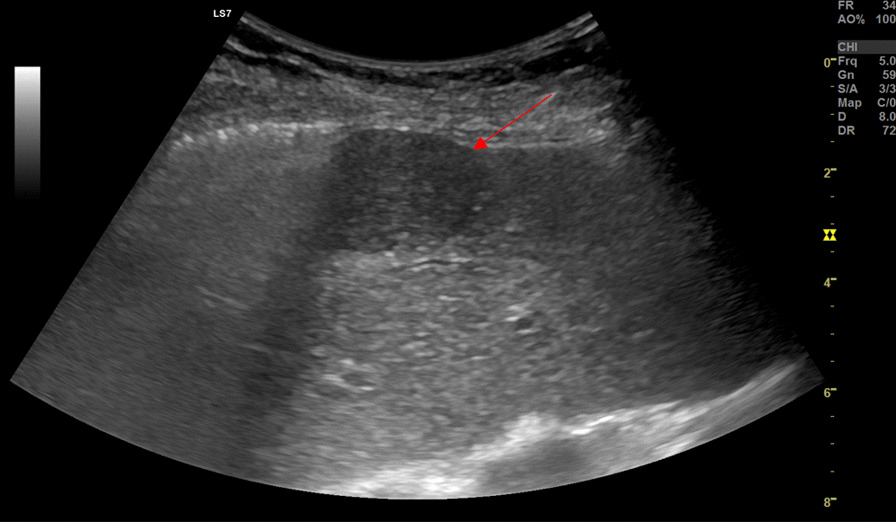
Abdominal B-Mode ultrasound of the hepatic subcapsular hypoechoic nodule. The arrow is pointing to the hepatic subcapsular hypoechoic nodule

At the moment of diagnosis (October 2020) the nodule had a diameter of 9/10 mm and was localized in the VI/VIIth hepatic segment. In June 2021 the abdominal ultrasound revealed the growth of the nodule to a diameter of 29 mm (Fig. 1).

**Fig. 2:**
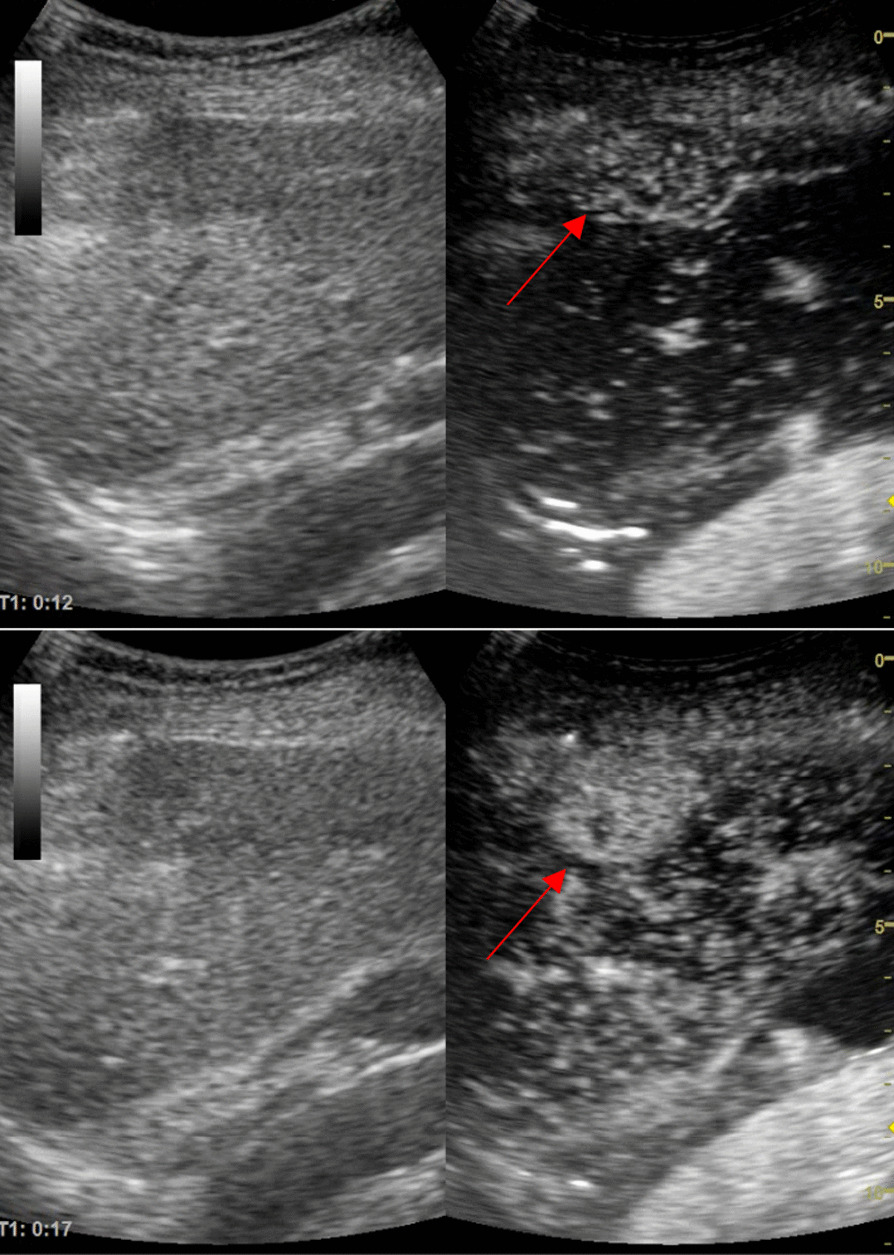
Sonovue® enhacement at 12 s (basket-like vessels are visible) and at 17 s the nodule enhanced almost completely. The upper arrow is pointing to the hepatic nodule at the beginning of the enhancement showing basket-like vessels. The lower arrow is pointing to the hepatic nodule showing the enhancement that is almost complete

**Fig. 3:**
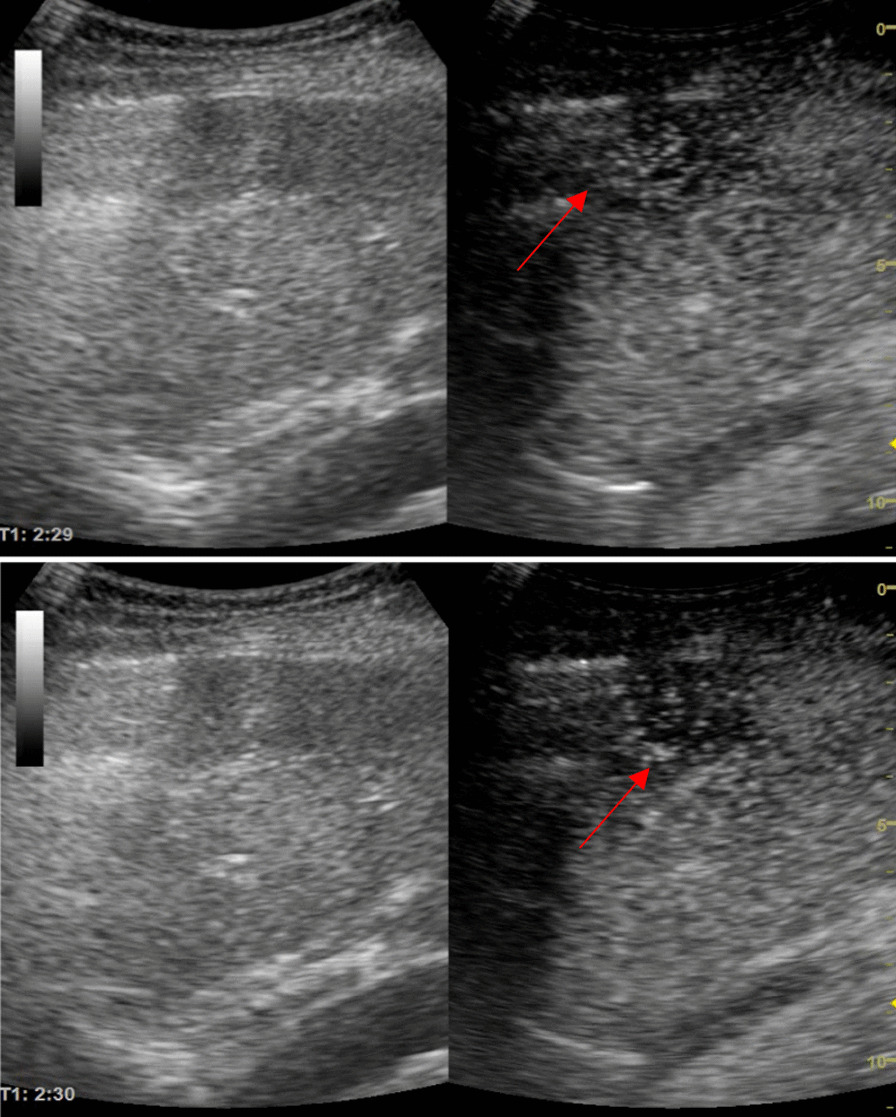
Late venous phase—incomplete washout at 2 min 29 s. The both arrows are pointing to the hepatic nodule showing the incomplete washout in the late venous phase

We decided to perform a contrast-enhanced ultrasound (CEUS) using SonoVue®. The enhancement started fast after contrast administration and was intense, from the periphery to the center of the nodule (Fig. 2). In the portal phase, the nodule remained slightly hypo-enhanced. In the venous phase (1 min and 20 s) the washout started, without a complete washout (Fig. 3). This perfusion pattern was considered specific for hepatocellular carcinoma (LI-RADS 5). Because the nodule was subcapsular, the biopsy was not an option due to the high risk of hemorrhage. Moreover, the Shear-Wave Elastography showed a liver stiffnes of 8.8 kPa, compatible with F3 fibrosis.

**Fig. 4:**
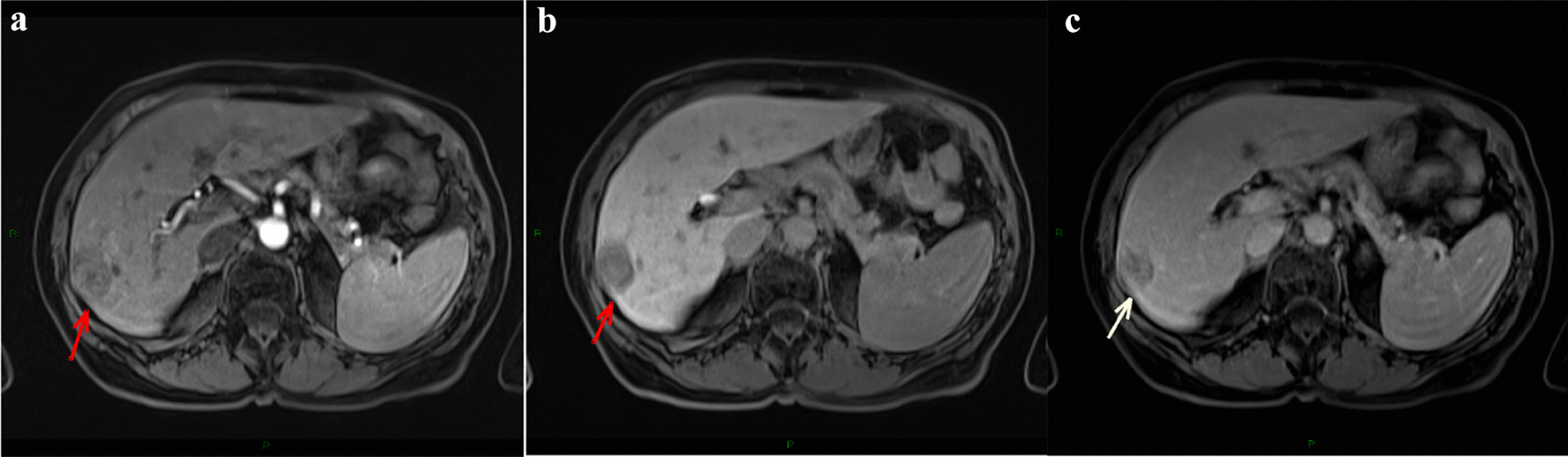
**a** T1 weighted sequence after gadoxetate disodium administration, arterial phase; **b** T1 weighted sequence, venous phase; **c** hepatobiliary phase—hypovascular, non-specific nodule. **a** The arrow is pointing to the hypointense hepatic nodule in the arterial phase after gadoxetate disodium administration. **b** The arrow is pointing to the hypointense hepatic nodule in the venous phase after gadoxetate disodium administration. **c** The arrow is pointing to the hypointense hepatic nodule in the hepatobiliary phase after gadoxetate disodium administration

Given the fact that AFP was slightly increased (10.8 ng/ml with UNV = 9 ng/ml), we also performed a liver MRI using gadoxetate disodium. On MRI the nodule was hypovascular, non-specific (Fig. 4), being classified as LI-RADS 3.

Treatment:

Our patient had liver cirrhosis and a unique liver nodule. However, the lesion was highly suggestive for HCC on CEUS (LI-RADS 5). A small percentage of hepatocellular carcinomas remain iso-/hypovascular after contrast administration on MRI, therefore we presumed that the nodule was HCC BCLC (Barcelona clinic liver cancer staging) Stage A. The patient was reffered to surgical resection. In July 2021 an atypical resection of the VIIth hepatic segment was performed.

**Fig. 5:**
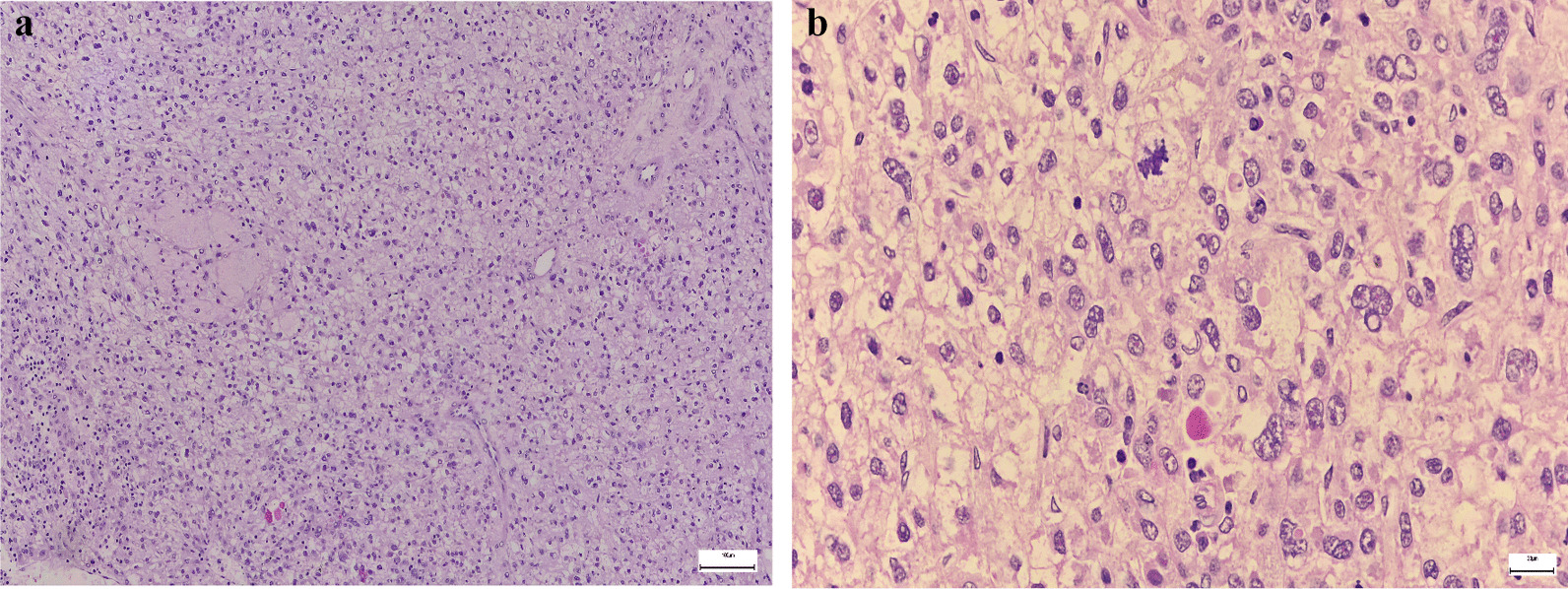
**a** Hepatocellular carcinoma, Hematoxylin and Eosin stain; **b** 40 × magnification

**Fig. 6:**
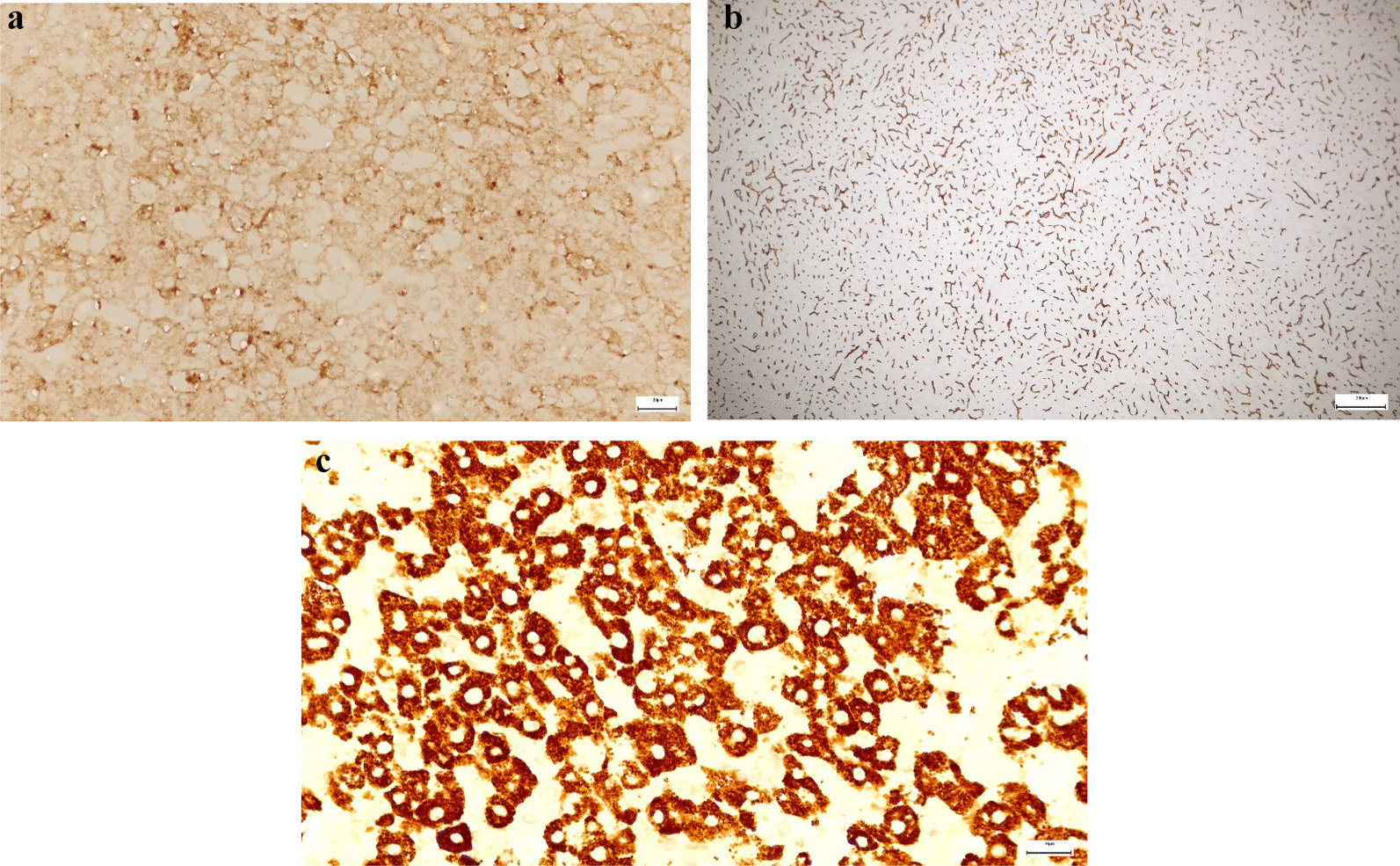
Immunohistochemistry: **a** Glypican3 positive cells; **b** CD34 positive cells; c. Hep Par-a positive cells

**Fig. 7:**
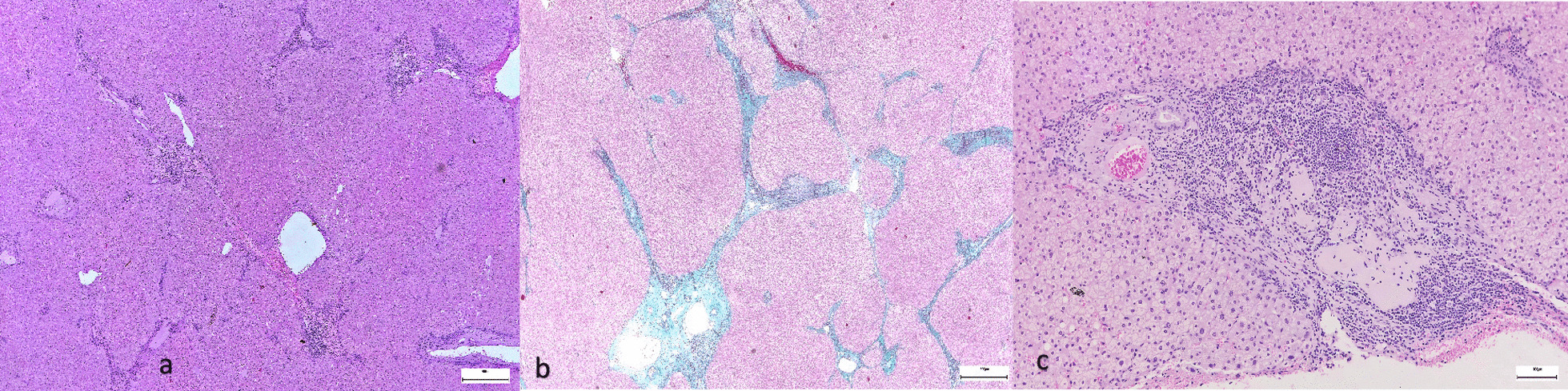
**a** Liver parenchyma (H&E stain); **b** Masson's trichrome stain showing porto-portal bridging fibrosis (Ishak 3); **c** Injury and inflammation of the bile ducts

Histological finding:

The histological examination revealed a moderately differentiated hepatocellular carcinoma (G2), pT2 Nx Mx L0 V1 R0, with predominant solid architecture of the tumor, without tumoral necrosis (Fig. 5a, b) and imunohistochemistry staining was performed (Hep Par-a positive, Glypican3 positive, CD34 positive as a marker of liver sinusoidal capillarization—Fig. 6a–c). The liver capsule was intact. Also, a lymph node was resected but no malignant cells were found at the histological examination. The histological exam of the liver parenchyma also revealed porto-portal bridging fibrosis, interpreted as Ishak 3 (Fig. 7a, b), which corresponds to F2 Metavir. Figure 7c shows the presence of inflammation and injury of the bile ducts, that are suggestive for PBC.

### Final diagnosis

After the histological examination and imunohistochemistry we could establish the final diagnosis: Hepatocellular carcinoma (G2), pT2 N0 M0 L0 V1 R0, BCLC Stage A. The tumor was under 5 cm so it was classified as T2, N0 shows that there were no regional lymph node metastasis and M0 shows that there were no distant metastasis. The tumor was moderately differentiated (G2).

L0: no lymphatic invasion; V1: venous invasion; R0: complete tumor resection with surgical margin microscopically negative for residual tumor.

### Outcome and follow-up

The patient was followed up once in our service 5 months after the surgery (December 2021). We performed an abdominal ultrasound that showed a surgical triangular scar in the VIIth hepatic segment. The surgical scar appeared to be hyperechoic and had a diameter of 35/21 mm. The liver was inhomogeneous, typical for liver cirrhosis. No hepatic nodules were found on the ultrasound examination. Blood analysis revealed nitrogen retention, the bilirubin levels were slightly increased, a normal platelet count, a normal hemoglobin level and a normal AFP level. The patient was also followed in an oncology service 6 months after the surgery and performed a computed tomography (CT) scan (January 2022) that showed no sign of tumor recurrence.

Table 2 summarizes the most important details of the last 3 presentations the patient had in our department.

**Table 2:** Nodule dimension, symptoms and AFP level at diagnosis and follow up

Date	Nodule dimension	Symptoms	AFP level (ng/ml)
October 2020	9/10 mm	None	Normal
June 2021	29 mm	None	10.8
December 2021 (follow-up)	–	None	Normal

## Discussion

The majority of HCCs are associated most frequently with chronic B or C viral hepatitis, alcohol intake or aflatoxin exposure. Cirrhosis is a strong risk factor associated with HCC. The causes of liver cirrhosis are chronic viral hepatitis, alcohol intake, metabolic diseases (NAFLD), hemocromathosis, alfa 1 antitrypsisn deficiency. All aetiologic forms of cirrhosis are at risk to be complicated by HCC formation, but the risk is higher for patients diagnosed with chronic viral hepatitis [2]. A recent meta-analysis showed a considerable increase in HCC incidence in a variety of liver diseases, including HCV, HBV chronic hepatitis, PBC, AIH, non-alcoholic fatty liver disease (NAFLD), when the disease progresses to the cirrhotic stage [8]. Even though AIH is a cause of HCC, several studies confirmed a lower risk (< 3%) of HCC in these patients compared to patients with other chronic liver diseases [9]. The risk factors for HCC development in patients with AIH are cirrhosis (the most significant), older age, male sex, alcohol consumption, and signs of portal hypertension [8]. Another meta-analysis performed on 29 studies including 22,615 patients reported an incidence rate of 4.17 per 1000 patient-years (95% CI: 3.17–5.47) for HCC in patients diagnosed with PBC. The meta-analysis also showed that the incidence of HCC in cirrhotic patients with PBC was of 15.7 per 1000 patient-years (95% CI: 8.73–28.24) compared to 2.68 per 1000 person-years in non-cirrhotic patients. The results confirmed that cirrhosis is a strong risk factor for HCC in patients with PBC [10]. Also, a meta-analysis of 25 studies determined the incidence of HCC in patients diagnosed with AIH with or without cirrhosis. The pooled incidence of HCC in patients diagnosed with AIH without cirrhosis was 3.06 per 1000 person-years (95% CI of 2.22–4.23) with a moderate-heterogeneity between studies. The pooled incidence of HCC in patients diagnosed with AIH with cirrhosis was 10.07 per 1000 person-years (95% CI 6.90–14.70) with a low heterogeneity between studies [11].

Both AIH and PBC progress in time to liver cirrhosis. Our patient was already in the cirrhotic stage at the time of the first admission in our department. The CEUS aspect of the HCC was rather typical, but given the slightly increased AFP level, MRI was performed. The MRI aspect of the tumor was atypical. The MRI characteristics of an HCC on T1 weighted sequence vary from hyper- to hypointense compared to the surrounding liver [12]. In our case the tumor was hypointense compared to the surrounding liver. After the administration of gadoxetate disodium the enhancement occurs usually in the arterial phase due to the tumor’s vascularity and the tumor is hyperintense compared to the surrounding liver. The hepatocellular carcinoma’s main supply comes from the hepatic artery rather than the portal vein and this leads to a rapid washout in the venous phase [13]. In this case the tumor remained hypointense compared to the surrounding liver after the administration of contrast. One explanation could be that early hepatocellular carcinomas can show hypo-/iso-enhancement on arterial-phase imaging, as a result of incomplete arterial neovascularization [12].

Before the administration of gadoxetate disodium the lesion appears hypointense compared to the rest of the parenchyma. After gadoxetate disodium administration on a T1 weighted sequence it is observed that the lesion remained hypointense (hypoenhanced) compared to the rest of the parenchyma in all vascular phases. In order to consider a nodule as HCC (LI-RADS 5) on a cirrhotic liver, the nodule has to be hyperenhanced in the arterial phase and one additional diagnostic criteria, either wash-out, or a capsule enhancing in the late phase or the increase in size of more than 50% in 6 month or less, has to be met. A small percentage of hepatocellular carcinomas can be isoenhanced and even hypoenhaced in the arterial phase compared to the rest of the parenchyma. Most of HCC nodules do not enhance in the hepatobiliary phase [14, 15].

Regarding the prognostic and survival, a study included patients that underwent surgical resection of HCC between 1998 and 2017 from an international multi institutional data base and had the purpose to observe the prognosis of the patients after HCC resection. The five-year OS (overall survival) for patients diagnosed with HCC BCLC stage A was 69.0% [16].

We also have to take into account that microscopic venous invasion was identified. Microscopic vascular invasion is a powerful predictor of poor overall survival (OS) after liver resection in patients with HCC. A study conducted by Kuo *et al.* calculated the 5‐year OS rate for the patients diagnosed with HCC that underwent liver resection and had no vascular invasion (NVI), microvessel invasion (MI) or microscopic portal vein invasion (MPVI). The 5-year OS rate was 87.2% in the NVI group, 83.3% in MI group, and 59.2% in the MPVI group. Overall survival in the MPVI group was significantly shorter than in the MI group and NVI group but there was no significant difference of OS between the NVI and MI groups [17]. In our case the patient was in the MI group so the 5-year OS rate is 83.3%.

The atypical resection of the VIIth hepatic segment had the aim to remove the tumor, obtain negative surgical margins and preserve as much liver parenchyma as possible. The anatomic liver resection has the advantage of removing potential undetectable satellite metastases and it is proven to be superior to non-anatomic (atypical resection). But the atypical resection offers a better liver function after surgery. This is an important aspect especially in patients with impaired liver function (for example liver cirrhosis) [18].

A particularity of this case is the fact that our patient was diagnosed with liver cirrhosis based on a histological exam of a liver biopsy performed in 2014 and in 2021 the liver parenchyma included in the resection piece showed fibrosis classified as Ishak 3 (F2 Metavir). It is already known that there are many factors that impact the accuracy of fibrosis staging in liver biopsy, for example, the lenght of the biopsy and the number of portal spaces. An adequate biopsy has to measure at least 15 mm (ideal 25 mm) and have at least 5 portal spaces. The etiology of chronic liver disease may also impact the liver biopsy. Moreover, the location of the biopsy is also important. A study showed that fibrosis stage between the right and the left lobe can vary up to 2 stages or even more. Last but not least there is also significant interobserver or intraobserver variability [19]. In our patient’s case the lenght of the biopsy in 2014 was greater than the one included in the resection piece in 2021 and the histological exams were performed by different observers.

## Conclusion

Our patient was a 71 years old female with several autoimmune disorders, without associated chronic viral hepatitis, with platelets count over 100 × 10^9^/L, without oesophageal varices or alcohol intake. When first described, the nodule could have been a regenerative nodule, but the dimensions increasing in time, the aspect on CEUS were in favour of HCC. The slightly increased AFP level, the atypical aspect of the nodule on MRI with gadoxetate disodium and the absence of other risk factors, except for the liver cirrhosis, were arguments against the diagnosis of HCC. These aspects represent the particularities of this case.

## Data Availability

The data that support the findings of this case report are available from Medical Records Department of the Second Medical Department of Internal Medicine at the County Emergency Hospital Cluj-Napoca and the Regional Institute of Gastroenterology and Hepatology Cluj-Napoca, but restrictions apply to the availability of these data, which were used under license for this case report and are not publicly available. Data are available from the authors with permission of Medical Records Department of the Second Medical Department of Internal Medicine at the County Emergency Hospital Cluj-Napoca and the Regional Institute of Gastroenterology and Hepatology Cluj-Napoca.
